# Editorial: Insomnia: A Heterogenic Disorder Often Comorbid With Other Disorders and Diseases

**DOI:** 10.3389/fpsyg.2021.758189

**Published:** 2021-09-14

**Authors:** Susanna Jernelöv, Ståle Pallesen, Bjørn Bjorvatn

**Affiliations:** ^1^Centre for Psychiatry Research, Department of Clinical Neuroscience, Karolinska Institutet, and Stockholm Health Care Services, Region Stockholm, Huddinge Hospital, Stockholm, Sweden; ^2^Division of Psychology, Department of Clinical Neuroscience, Karolinska Institutet, Stockholm, Sweden; ^3^Department of Psychosocial Science, University of Bergen, Bergen, Norway; ^4^Norwegian Competence Center for Sleep Disorders, Haukeland University Hospital, Bergen, Norway; ^5^Department of Global Public Health and Primary Care, University of Bergen, Bergen, Norway

**Keywords:** chronic insomnia, cognitive behavioral therapy, comorbidities, prevalence, prediction, shift work, subtypes, suicidal ideation

Chronic insomnia disorder is defined as subjective complaints of problems initiating or maintaining sleep, causing negative daytime consequences, for a duration of at least 3 months (American Psychiatric Association, 2013; American Academy of Sleep Medicine, 2014). The prevalence of both acute and chronic insomnia symptoms in the general population is high, and depends on age, sex and socioeconomic status (Riemann et al., 2017), and insomnia is commonly comorbid with psychological and somatic disorders (Riemann et al., 2017). There is a strong association between insomnia and psychopathology (Hertenstein et al., 2019), and an increasing number of studies suggest that treatment targeting insomnia also improve mood symptoms (Blom et al., 2015; Ballesio et al., 2018; Ho et al., 2020). Although insomnia disorder is highly prevalent, it is seldom adequately assessed and treated (Grandner and Chakravorty, 2017).

In this Research Topic, insomnia is illuminated from different angles, and a goal has been to evaluate and disentangle the bidirectional associations and interactions between insomnia and other psychological and somatic conditions. Bjorvatn et al. provide a narrative review of diagnostic and treatment challenges of insomnia disorder in relation to other sleep disorders, with the aim to present current clinical practice and stimulate to more research into diagnostic processes and bidirectional associations.

Sivertsen et al. used a large sample of young adults (18–35 years) to investigate prevalence of insomnia, sleep duration, and their relation to mental and physical disorders. In line with previous epidemiological research (e.g., Hertenstein et al., 2019), they show in their study comprising 50,000 participants, that Insomnia according to DSM-5 criteria and short sleep duration (<7.5 h) are strongly associated with a range of different disorders and conditions, and most strongly so with mental disorders.

Separating insomnia disorder from other sleep disorders is of great importance, as treatment-of-choice varies substantially between different sleep disorders. For insomnia, treatment-of-choice is cognitive behavioral therapy for insomnia (CBT-I). Recent research has shown that treating insomnia with CBT-I is both feasible and effective when insomnia is comorbid with other disorders (Wu et al., 2015). In their paper, Sweetman et al. investigated predictors of treatment response to CBT-I in patients with comorbid insomnia and sleep apnea (COMISA). Interestingly, their results show that higher OSA severity predicted a greater treatment-response to CBT-I. The authors conclude that people with COMISA should be treated with CBT-I, as it is effective even in the presence of severe OSA and objective sleep disturbance.

The relation between Insomnia and OSA is also the subject in the study by Lundetræ et al. In a group of patients with OSA receiving CPAP treatment, they investigated the effect of CPAP treatment on insomnia symptom severity, and found that insomnia symptoms do improve following CPAP treatment. Furthermore, the improvements in insomnia symptoms were larger in patients who adhered (4+ h/night) compared to those who did not adhere (<4 h/night) to the CPAP treatment.

It is well-established that insomnia is a risk factor for poor mental health (e.g., Ford and Kamerow, 1989) including depression and suicidal ideation (e.g., Pigeon et al., 2012b; Johansson et al., 2021), and previous research has shown that the risk of completed suicide is increased in veterans with disturbed sleep (Pigeon et al., 2012a). In a sample of regular psychiatric patients seeking digital treatment for insomnia, Jernelöv et al. investigated the prevalence of suicidal ideation, and found that levels of suicidal ideation were surprisingly low, with only 1% seeing suicide as “a way out,” and more than 80% endorsing a normal or near normal appetite for life before treatment. Interestingly, despite these low initial levels, but in line with studies on more severely depressed and suicidal patients (Trockel et al., 2015; Pigeon et al., 2019), suicidal ideation decreased further following treatment with CBT-I, implying a potential for CBT-I to play a role in the prevention of suicide.

Holzinger et al. investigated insomnia symptoms and total sleep time in non-shift workers, regular shift workers and irregular shift workers employed by a railway company. They found that the sleep of irregular shift workers was of poor quality, though not necessarily to the point of clinical insomnia, and that working regular shifts was associated with the best sleep quality and the longest sleep duration. They also investigated the impact of the Big Five personality traits and found for instance that agreeableness and perfectionism increased the effect of work schedule on total sleep time.

With the aim to identify relevant predictors and risk-factors for developing insomnia, Norell-Clarke et al. investigated whether sleep-related cognitive processes such as safety behaviors and somatic arousal could predict new cases of insomnia. The results suggest that an elevated degree of safety behaviors and heightened pre-sleep arousal not only maintain insomnia but also constitute risk factors for developing insomnia. With partial support for the Cognitive Model of Insomnia, the results provide interesting openings for future research into screening and prevention of insomnia.

Several recent studies suggest that insomnia is a heterogenic disorder with different phenotypes (e.g., Blanken et al., 2019), hence identifying clinically relevant subtypes could be of great importance. To this end, Fietze et al. used a cohort of patients at a sleep specialist clinic. They found that their cohort displayed a long history of suffering and that the sleep specialist clinic was usually not the first point of contact. They therefore suggest new questions (e.g., ability to fall asleep during the day, effects of non-medical therapy methods, symptom stability) to include in anamnesis to help differentiate between insomnia subtypes and better understand the severity of insomnia, and to individualize therapy.

Together, the papers in this Research Topic contribute with new insights to drive research on insomnia in novel directions, with the ultimate goal to alleviate suffering in patients with this very common and debilitating condition.

## Author Contributions

SJ: drafting the paper. SP and BB: critical review of the paper. All authors have read and approved the final version of the manuscript.

## Conflict of Interest

The authors declare that the research was conducted in the absence of any commercial or financial relationships that could be construed as a potential conflict of interest.

## Publisher's Note

All claims expressed in this article are solely those of the authors and do not necessarily represent those of their affiliated organizations, or those of the publisher, the editors and the reviewers. Any product that may be evaluated in this article, or claim that may be made by its manufacturer, is not guaranteed or endorsed by the publisher.
